# Optimal design and control of an electromechanical transfemoral prosthesis with energy regeneration

**DOI:** 10.1371/journal.pone.0188266

**Published:** 2017-11-17

**Authors:** Farbod Rohani, Hanz Richter, Antonie J. van den Bogert

**Affiliations:** 1 Department of Electrical and Computer Engineering, Cleveland State University, Cleveland, Ohio, United States of America; 2 Department of Mechanical Engineering, Cleveland State University, Cleveland, Ohio, United States of America; Chongqing University, CHINA

## Abstract

In this paper, we present the design of an electromechanical above-knee active prosthesis with energy storage and regeneration. The system consists of geared knee and ankle motors, parallel springs for each motor, an ultracapacitor, and controllable four-quadrant power converters. The goal is to maximize the performance of the system by finding optimal controls and design parameters. A model of the system dynamics was developed, and used to solve a combined trajectory and design optimization problem. The objectives of the optimization were to minimize tracking error relative to human joint motions, as well as energy use. The optimization problem was solved by the method of direct collocation, based on joint torque and joint angle data from ten subjects walking at three speeds. After optimization of controls and design parameters, the simulated system could operate at zero energy cost while still closely emulating able-bodied gait. This was achieved by controlled energy transfer between knee and ankle, and by controlled storage and release of energy throughout the gait cycle. Optimal gear ratios and spring parameters were similar across subjects and walking speeds.

## Introduction

Walking with a transfemoral prosthesis requires up to 65% more energy than able-bodied walking [1–3], which mostly arises from the loss of knee function during the stance phase of gait [3]. The increased energy cost suggests excessive compensatory muscle actions, which may be responsible for adverse health conditions in amputees, such as osteoarthritis [4, 5]. Conventional prosthetic knees are controlled dampers which cannot generate positive work at any time, and therefore do not replicate able-bodied muscle function. Studies show that recent computer-controlled dampers have only reduced the energy cost by 3%-5% [6, 7], compared to a passive mechanical knee. Active, motorized prostheses have the potential to overcome this limitation. For instance, a powered ankle prosthesis was shown to reduce the metabolic energy cost of walking, as well as improve the quality of gait [8]. A powered knee-ankle prosthesis has recently been described [9] and has the potential to fully replicate able-bodied muscle function in transfemoral amputees. However, powered prostheses consume energy and a large battery is required to allow a full day of activity without recharging [10, 11].

The efficiency of active prostheses can be improved by energy regeneration. During normal walking, there are periods of the gait cycle when knee, ankle or both perform negative work [12]. This negative work could be transferred to the other joint, or stored and used at a different time in the gait cycle. A hydraulic knee model with mechanical energy storage was optimized for walking, running, and a sit-stand-sit cycle [13]. Energy regeneration has been successfully used in active ankle joints [14–17]. Recently, Unal developed a prosthetic leg that can transfer energy between knee and ankle [18]. An electromechanical knee with energy regeneration was presented in [19–22]. In electromechanical systems, negative work can be transferred to a battery for later use. However, batteries cannot meet the high charging rate demanded to absorb the large bursts of negative power that occur during the gait cycle. Unlike batteries, ultracapacitors are highly efficient and can be charged and discharged quickly [23]. An electromechanical knee joint with ultracapacitor was modeled by Warner et al. [21]. Multi-objective optimization was used to optimize the mechanical design as well as an impedance controller [21]. In a two-joint system, the need for electrical energy storage can be reduced by transferring energy directly between joints [24], at times when one joint requires negative work and the other requires positive work.

**Fig 1:**
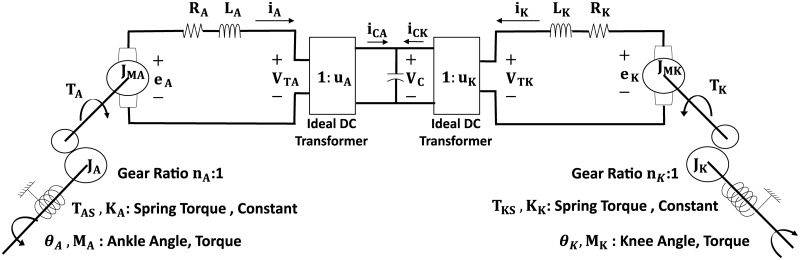
Schematic diagram of the electromechanical prosthetic system with motors at ankle (left) and knee (right).

In this paper, we present an electromechanical knee-ankle prosthesis which can transfer energy between joints and includes an ultracapacitor for storage. The energy flow between capacitor and motors is controlled by two four-quadrant power converters. Parallel springs are used to assist the motors (Fig 1). Specific goals of this work are: (1) to find optimal design parameters and control strategies, and (2) to predict how well the system will perform when operating without an external energy supply. In order to achieve these goals, we develop a dynamic model of the proposed device, and then use optimal control techniques to optimize simultaneously the state and control trajectories of the system, and its design parameters.

## System model

The prosthesis system consists of geared motors, springs, power converters, and a capacitor (Fig 1). The knee and ankle are each actuated by a 24 V DC motor. The Pittmann 14201 series motor was selected because it has suitable size and weight for this application. Armature resistance *R*, motor torque constant *α*, and inertial parameters *J* were obtained from the manufacturer’s specifications. An ideal gearbox was assumed between each motor and the joint. A linear spring was placed across each joint to potentially assist the motor. Gear ratios *n* and spring parameters will be found by design optimization. Power is supplied by an ultracapacitor with capacitance *C* = 50 F. The voltage supplied to each motor is controlled by a four-quadrant power converter between capacitor and motor. The power converters were modeled as DC transformers with voltage ratios *u*_*k*_ and *u*_*a*_ as control inputs.

The mechanical inputs of the system are the externally applied knee torque (*M*_*k*_) and the ankle torque (*M*_*a*_) which are given as a function of time. Table 1 lists the system parameters, and the equations for all system components are provided in Table 2. Components were modeled using standard equations for DC motors and other components [25]. The power converter model initially assumes an efficiency of 100%. Energy loss will be included later.

**Table 1:** The knee and ankle parameters in the prosthetic system.

parameter	knee	ankle	value	unit
gear train inertia	*J*_*k*_	*J*_*a*_	1.29*e*^−5^	kg m^2^
rotor inertia	*J*_mk_	*J*_ma_	1.13*e*^−5^	kg m^2^
motor constant	*α*_*k*_	*α*_*a*_	0.053	NmA^−1^
armature resistance	*R*_*k*_	*R*_*a*_	2.79	Ω
capacitance	*C*	*C*	50	F
armature inductance	*L*_*k*_	*L*_*a*_	0.00254	H
spring constant	*k*_*k*_	*k*_*a*_	optimized	Nm/rad^−1^
spring resting angle	*ϕ*_0*k*_	*ϕ*_0*a*_	optimized	rad
voltage control	*u*_*k*_(*t*)	*u*_*a*_(*t*)	optimized	
gear ratio	*n*_*k*_	*n*_*a*_	optimized	
joint torque	*M*_*k*_(*t*)	*M*_*a*_(*t*)	from gait data	Nm

**Table 2:** The dynamic and algebraic equations for components of the system. Parameters and inputs were defined in Table 1. Other system variables are: joint angle *ϕ*, spring torque *T*_*S*_, motor torque *T*, motor current *i*, capacitor voltage *V*, capacitor current *i*_*C*_, power converter output voltages *V*_*T*_. The inductance *L* of the motors was neglected.

description	knee equation	ankle equation
motor dynamics	$M_{k}-T_{S,k}-n_{k}T_{k}=\left(J_{k}+n_{k}^{2}J_{mk}\right){\ddot{\phi }}_{k}$	$M_{a}-T_{S,a}-n_{A}T_{a}=\left(J_{a}+n_{a}^{2}J_{ma}\right){\ddot{\phi }}_{a}$
spring torque	*T*_*S*,*k*_ = *k*_*k*_(*ϕ*_*k*_ − *ϕ*_0*k*_)	*T*_*S*,*a*_ = *k*_*a*_(*ϕ*_*a*_ − *ϕ*_0*a*_)
motor torque	*T*_*k*_ = *α*_*k*_ *i*_*k*_	*T*_*a*_ = *α*_*a*_ *i*_*a*_
motor back-emf	$e_{k}=\alpha_{k}n_{k}{\dot{\phi }}_{k}$	$e_{a}=\alpha_{a}n_{a}{\dot{\phi }}_{a}$
Kirchhoff’s voltage law	*e*_*k*_ = *R*_*k*_ *i*_*k*_ + *V*_*T*,*k*_	*e*_*A*_ = *R*_*a*_ *i*_*a*_ + *V*_*T*,*a*_
capacitor dynamics	$C\dot{V}=i_{C,k}+i_{C,a}$	
voltage converter	*V*_*T*,*k*_ = *u*_*k*_ *V*	*V*_*T*,*a*_ = *u*_*a*_ *V*
current converter	*i*_*C*,*k*_ = *u*_*k*_ *i*_*k*_	*i*_*C*,*a*_ = *u*_*a*_ *i*_*a*_

The five state variables of the dynamic system are capacitor voltage, knee and ankle angles, and knee and ankle angular velocity:

$$\begin{array}{c} x={\left(\phi_{k},{\dot{\phi }}_{k},V,\phi_{a},{\dot{\phi }}_{a}\right)}^{T} \end{array}$$ (1)

Two control inputs control the voltage ratios of the power converters:

$$\begin{array}{c} u={\left(u_{k},u_{a}\right)}^{T} \end{array}$$ (2)

From the component equations in Table 2, we can derive five coupled differential equations for the system dynamics:

$$\begin{array}{c} \dot{x_{1}}-x_{2}=0 \end{array}$$ (3)

$$\begin{array}{c} (J_{k}+n_{k}^{2}J_{mk})\dot{x_{2}}+k_{k}\left(x_{1}-\phi_{0k}\right)-M_{k}\left(t\right)+\frac{\alpha_{k}n_{k}(\alpha_{k}n_{k}x_{2}-u_{1}x_{3})}{R_{k}}=0 \end{array}$$ (4)

$$\begin{array}{c} C\dot{x_{3}}+\frac{u_{1}(u_{1}x_{3}-\alpha_{k}n_{k}x_{2})}{R_{k}}+\frac{u_{2}(u_{2}x_{3}-\alpha_{a}n_{a}x_{5})}{R_{a}}=0 \end{array}$$ (5)

$$\begin{array}{c} \dot{x_{4}}-x_{5}=0 \end{array}$$ (6)

$$\begin{array}{c} (J_{a}+n_{a}^{2}J_{ma})\dot{x_{5}}+k_{a}\left(x_{4}-\phi_{0a}\right)-M_{a}\left(t\right)+\frac{\alpha_{a}n_{a}(\alpha_{a}n_{a}x_{5}-u_{2}x_{3})}{R_{a}}=0 \end{array}$$ (7)

The system dynamics model is now available as an implicit differential equation:

$$\begin{array}{c} f(x,\dot{x},u,t,p)=0, \end{array}$$ (8)

where **p** is a vector containing the design parameters that must be optimized:

$$\begin{array}{c} p={\left(n_{k},n_{a},k_{k},k_{a},\phi_{0k},\phi_{0a}\right)}^{T} \end{array}$$ (9)

The next step is to find, simultaneously, the controls **u**(*t*) and parameters **p** that optimize the performance of the system.

## Optimization

In this section we describe the optimal control methods to find the state trajectories, control trajectories and hardware design parameters simultaneously. The objective is to minimize the loss of stored energy in the capacitor and to minimize the tracking error between the simulated joint motions and human gait. The cost function was defined as a weighted sum of these objectives, for a movement of duration *T*:

$$\begin{array}{c} \;F=\frac{W_{1}}{T}\;\int_{0}^{T}\left[{\left(\frac{\phi_{k}\left(t\right)-\phi_{k,d}\left(t\right)}{\sigma_{k}}\right)}^{2}+{\left(\frac{\phi_{a}\left(t\right)-\phi_{a,d}\left(t\right)}{\sigma_{a}}\right)}^{2}\right]dt+\frac{W_{2}}{2}\left(V{\left(0\right)}^{2}-V{\left(T\right)}^{2}\right) \end{array}$$ (10)

In the first term of the objective, the differences between simulated joint angles *ϕ*(*t*) and reference angles *ϕ*_*d*_(*t*) was divided by the standard deviation of the reference signal to make tracking error a dimensionless relative measure. Reference angles *ϕ*_*d*_(*t*), and corresponding joint torques *M*(*t*) were obtained from ([26]) for 10 subjects, four females and six males between 63 kg and 90.7 kg, each walking at slow speed (0.8 m/s), normal speed (1.2 m/s), and fast speed (1.6 m/s). In the second term, energy loss was computed from the capacitor voltage at the beginning and at the end of the gait cycle. The weight factors *W*_1_ and *W*_2_ can be chosen to emphasize the importance of each term in the cost function.

We will optimize one gait cycle, and will therefore require that the joint motions are periodic:

$$\begin{array}{c} \phi_{k}\left(T\right)-\phi_{k}\left(0\right)=0 \end{array}$$ (11)

$$\begin{array}{c} {\dot{\phi }}_{k}\left(T\right)-{\dot{\phi }}_{k}\left(0\right)=0 \end{array}$$ (12)

$$\begin{array}{c} \phi_{a}\left(T\right)-\phi_{a}\left(0\right)=0 \end{array}$$ (13)

$$\begin{array}{c} {\dot{\phi }}_{a}\left(T\right)-{\dot{\phi }}_{a}\left(0\right)=0 \end{array}$$ (14)

The optimal control problem is now to find a state trajectory **x**(*t*), control trajectory **u**(*t*), and parameter vector **p**, such that the cost function (10) is minimized, the dynamics Eq (8) are satisfied, and periodicity constraints (11–14) are satisfied. This problem was transcribed into a large-scale nonlinear program (NLP) using direct collocation (DC) [[13, 27]]. The trajectories were discretized on a temporal mesh of *N* nodes. A vector **X** of unknowns was defined by stacking the states and controls at these time points, and the parameter vector, into one column vector:

$$\begin{array}{c} X={\left(x_{1}^{T},u_{1}^{T},\ldots ,x_{N}^{T},u_{N}^{T},p^{T}\right)}^{T} \end{array}$$ (15)

After discretization, the integration over the period *T* in (10) is replaced by a summation over the nodes. The dynamics Eq (8) were transformed into 5(*N* − 1) algebraic constraints by the Midpoint Euler method:

$$\begin{array}{c} \;f(\frac{x_{i+1}+x_{i}}{2},\frac{x_{i+1}-x_{i}}{t_{i+1}-t_{i}},\frac{u_{i+1}+u_{i}}{2},\frac{t_{i+1}+t_{i}}{2},p)=0\;\text{for}\;i=1\ldots N-1 \end{array}$$ (16)

The NLP was solved by IPOPT [28], version 3.11.0, with **X** = **0** as initial guess. The objective function and constraints, and their analytical gradients were coded in Matlab (version 2016a). Mesh refinement was performed and it was found that the results at 50 and 100 time nodes were virtually identical, so *N* = 100 was used for all optimizations. Capacitor voltage at the start of the gait cycle was set to 8 V.

After each optimization, the root-mean-square (RMS) tracking errors were computed:

$$\begin{array}{c} RMS_{\text{knee}}=\sqrt{\frac{1}{N}\sum_{i=1}^{N}{\left(\phi_{k,i}-\phi_{kd,i}\right)}^{2}} \end{array}$$ (17)

$$\begin{array}{c} RMS_{\text{ankle}}=\sqrt{\frac{1}{N}\sum_{i=1}^{N}{\left(\phi_{a,i}-\phi_{ad,i}\right)}^{2}} \end{array}$$ (18)

Mechanical work delivered by the motors was calculated by integrating the product of angular velocity and torque:

$$\begin{array}{c} W_{\text{knee}}=\int_{0}^{T}{\dot{\phi }}_{k}\left(t\right)M_{k}\left(t\right)dt \end{array}$$ (19)

$$\begin{array}{c} W_{\text{ankle}}=\int_{0}^{T}{\dot{\phi }}_{a}\left(t\right)M_{a}\left(t\right)dt \end{array}$$ (20)

Total motor heat output was calculated by integrating the product of current and voltage across the resistors:

$$\begin{array}{c} H_{\text{motors}}=\int_{0}^{T}(R_{k}i_{k}^{2}\left(t\right)+R_{a}i_{a}^{2}\left(t\right))dt \end{array}$$ (21)

The model of system dynamics initially assumes ideal power converters. However, after optimization, the energy loss in the power converters was computed based on an efficiency of *η* = 90% [29]:

$$\begin{array}{c} H_{conv}=\left(1-\eta \right)\int_{0}^{T}(|V_{T,k}\left(t\right)i_{k}\left(t\right)|+|V_{T,a}\left(t\right)i_{a}\left(t\right)|)dt \end{array}$$ (22)

All integrals were computed from the discrete-time trajectories by the midpoint method, to be consistent with the discrete time approximation of the system dynamics.

The change in capacitor stored energy was calculated as:

$$\begin{array}{c} \Delta E=\frac{C}{2}(V{\left(T\right)}^{2}-V{\left(0\right)}^{2})-H_{conv} \end{array}$$ (23)

The second term accounts for the energy loss in the power converters.

Gait data from subject 1, walking at medium speed, was used to explore the optimization problem. A Pareto front was generated by solving the problem for a series of weight ratios *W*_1_/*W*_2_. Three cases on the Pareto front were selected for further analysis: case 1 with negative energy loss (i.e. energy gain) and poor tracking, case 2 with almost zero total energy loss, and case 3 with high energy loss and near-perfect tracking.

Case 2 is of special interest, because it is the case in which the change in stored energy is close to zero. This represents a system trajectory that could be performed without use of external energy. For this case, the detailed trajectories of joint angles, capacitor voltage, and control signals will be presented for this subject at all three speeds.

An exact zero-energy solution was then obtained from gait data from all subjects and all speeds, by adding the constraint Δ*E* = 0 to the optimization problem, and removing the second term from the cost function. The constraint was enforced iteratively. In the first optimization, the constraint equation converter loss was not included in the constraint equation. After calculating converter loss, this value was included in the constraint equation for the second optimization. This converged very quickly, and after a third optimization, Δ*E* = 0 was achieved within 0.1 J. Averages and standard deviations of performance measures and design parameters were calculated from these optimizations.

Matlab code and gait data are included as supplements (S1 File, S1 Data). Executing the code will generate the complete set of tables and figures that are presented in the Results section. A total of 105 optimizations was performed, 15 for the Pareto front, and three optimizations each to solve the zero-energy case for data from ten subjects walking at three speeds. Each optimization requires about 30 seconds of computation time, using Matlab 2016a (64-bit) and IPOPT 3.11.0 on the Windows 7 operating system with a 2.4 GHz processor.

## Results

**Fig 2:**
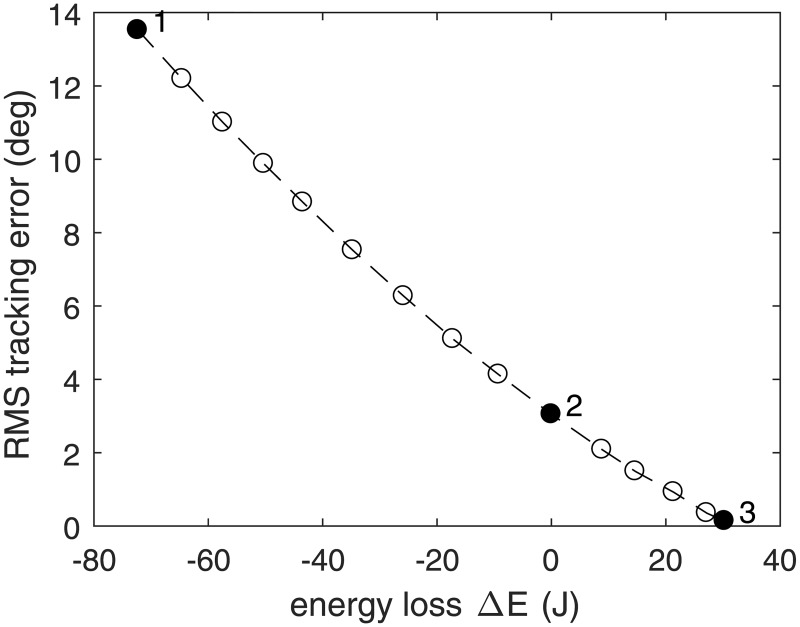
The tradeoff between energy cost and RMS tracking error shown as a Pareto front.

**Fig 3:**
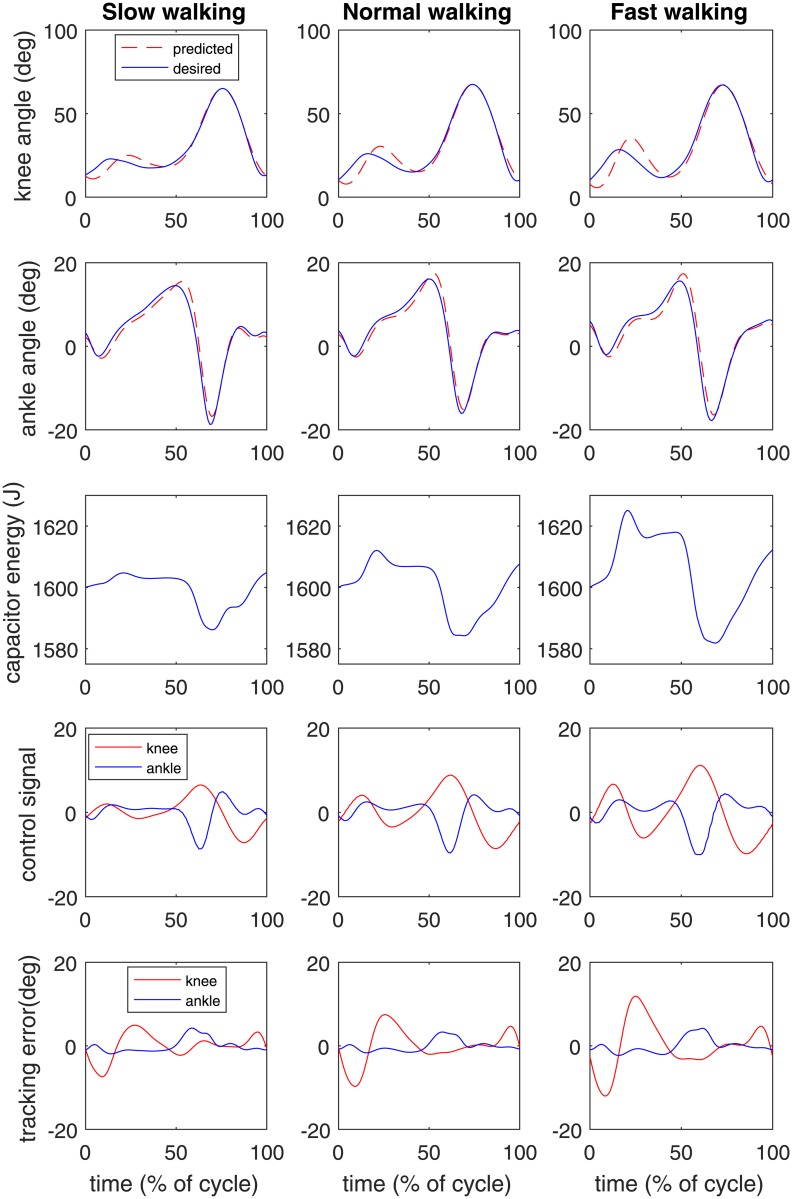
Optimal joint angles, capacitor energy, control signals, and tracking errors for three walking speeds in the zero-energy case for subject 1. Time is expressed as a percentage of the gait cycle, from heel strike to the next heel strike. See text for further details.

The trade-off between the tracking objective and the capacitor energy was explored for the gait data of Subject 1 walking at normal speed, resulting in a Pareto plot (Fig 2). Each point represents a specific combination of weighting factors. Three cases are indicated in (Fig 2), with details provided in Table 3. When tracking weight is low (case 1), about 70 J of net energy was harvested from the applied torques, but the tracking error was unacceptably large. When tracking weight was very high (case 3), the tracking error was nearly zero, but the system lost 30 J of energy during the cycle. In an intermediate solution (case 2), RMS tracking error was 4.1deg for the knee and 1.4deg for the ankle, which is an acceptable performance, and energy use was zero. The system trajectories for this case are presented in Fig 3, middle column. The joint angle trajectories show that the joint movements were subtly altered, relative to the recorded human motions, just enough to eliminate the need for external energy for the system.

**Table 3:** Work-energy values, optimized gear ratios and optimized spring parameters (stiffness and resting angle) for the three Pareto-optimal solutions based on gait data from subject 1 walking at normal speed.

	case 1	case 2	case 3
**RMS_knee_** (**deg**)	18.4	4.1	0.2
**RMS_ankle_** (**deg**)	5.1	1.4	0.0
**W_knee_** (**J**)	−80.0	−23.5	−6.6
**W_ankle_** (**J**)	−23.4	−2.1	5.1
**motor heat** (**J**)	18.0	17.6	23.6
**converter loss** (**J**)	13.0	8.1	8.3
**ΔE** (**J**)	−72.3	0.0	30.3
**n_knee_**	223.4	280.2	254.8
**n_ankle_**	229.7	246.2	250.3
**k_knee_** (**Nm**/**rad**)	83.7	81.3	72.8
**k_ankle_** (**Nm**/**rad**)	311.9	324.1	317.3
*ϕ*_**0**,**knee**_ (**deg**)	27.1	26.9	26.6
*ϕ*_**0**,**ankle**_ (**deg**)	−0.8	−0.6	−0.7

Table 3 shows the energy balance of the system in each of the three solutions, separated into the mechanical work delivered by the motors, the heat generated in the motors, the change in stored energy, and energy loss in the power converter. The optimal design parameters for the three cases are also included in Table 3.

Table 4 presents the optimization results for the zero-energy case at three speeds, averaged over the ten subjects. At higher speeds, the RMS tracking errors increase as well as the energy that is dissipated as heat in the motor and transformers. To compensate for the heat losses, energy is harvested from the joint motions, primarily from the knee. Optimal design parameters are not very sensitive to walking speed, with the exception of the knee spring stiffness. The complete system trajectories are shown for the Subject 1 optimizations in in Fig 3.

**Table 4:** The mean and standard deviation of optimized parameters and work-energy values for the prosthesis when using zero energy. Optimizations were done with gait data from ten subjects walking at different speeds.

	Slow	Normal	Fast
**RMS_knee_** (**deg**)	3.0 ± 1.0	4.6 ± 1.5	6.1 ± 1.5
**RMS_ankle_** (**deg**)	1.1 ± 0.4	1.5 ± 0.7	2.3 ± 1.0
**W_knee_** (**J**)	−9.6 ± 4.3	−26.2 ± 7.1	−47.5 ± 15.1
**W_ankle_** (**J**)	−4.9 ± 3.1	−2.6 ± 2.6	0.9 ± 3.5
**motor heat** (**J**)	9.6 ± 3.6	20.1 ± 4.5	33.4 ± 8.9
**converter loss** (**J**)	4.9 ± 1.5	8.6 ± 2.0	13.3 ± 4.0
**ΔE** (**J**)	0.0 ± 0.1	0.0 ± 0.1	0.0 ± 0.1
**n_knee_**	281 ± 48	268 ± 41	269 ± 41
**n_ankle_**	284 ± 28	268 ± 33	265 ± 45
**k_knee_** (**Nm**/**rad**)	70 ± 27	97 ± 24	134 ± 38
**k_ankle_** (**Nm**/**rad**)	345 ± 67	353 ± 77	370 ± 80
*ϕ*_**0**,**knee**_ (**deg**)	27 ± 3	28 ± 2	27 ± 2
*ϕ*_**0**,**ankle**_ (**deg**)	−3 ± 3	−3 ± 3	−4 ± 3

## Discussion

A regenerative electromechanical above-knee prosthesis for both knee and ankle was presented. We modeled the system with a DC motor, spring, gear and a controllable power converter for each knee and ankle, and an ultracapacitor to store and release the energy within the system. Based on the dynamic model of the proposed device, we used the direct collocation method to find optimal state and control trajectories as well as optimal design parameters. Results based on gait data from ten subjects with three different walking speeds showed that operating the system with zero energy is theoretically possible without unacceptable tracking error.

The principle of zero-energy operation can be understood by examining the system trajectories in Fig 3. The capacitor energy increases after heelstrike, when the knee absorbs mechanical energy in the K1 (weight acceptance) phase [12]. The capacitor loses energy rapidly between 50% and 60% of the gait cycle to generate active push-off in the ankle, and is subsequently recharged during the swing phase when the knee motor can absorb mechanical energy during the K3 and K4 phases [12]. Of particular interest is the knee control at the beginning of the gait cycle. The optimal control strategy delays the knee flexion slightly, compared to the desired normal gait, and this greatly reduces the energy cost of knee motion. A similar delay is seen in the ankle at the end of the stance phase, around 60% of the gait cycle (Fig 3). The tracking errors (Fig 3, bottom panels) show these strategies very clearly. The system deviates from the desired knee motion during weight acceptance, and from the desired ankle motion during push off. Tracking errors in the ankle are smaller, because the cost function was normalized to the motion magnitude *σ*, which is smaller in this joint. The tracking errors seem small enough, but it remains to be seen how these will affect the user’s walking performance.

It should be noted that the capacitor energy trajectory (Fig 3) shows a net increase during the cycle. This removes energy from the mechanical system, by the exact amount that would have happened due to 10% energy loss in the power converters. If energy loss in the power converters had been modeled, we would have had perfectly periodic capacitor energy trajectories. The energy loss model, however, involves an absolute value operator which is not twice differentiable. Despite many attempts, IPOPT was unable to solve the optimal control problem in the presence of this discontinuity. IPOPT estimates the Hessian matrix of the Lagrangian function, and this matrix goes to infinity when the first derivative of objective or constraints has a discontinuity. This occurs when the argument of the absolute value function is zero at any time point, which happens whenever there is a reversals of power flow in the power converters. To avoid this issue, we used an iterative approximation approach which produced correct performance predictions (zero-energy joint motions) and optimal design parameters for the non-ideal power converters, as can be verified by inspecting the dynamics equations. It must be kept in mind, however, that the capacitor voltage, and the power converter control signals that are presented here would be different in the true system. A similar approach would be possible if one wanted to account for frictional losses in the gear system.

In the simulations, externally applied joint torques were used as known inputs. These inputs were obtained from normal human gait data. During actual use, however, the user can adapt their gait and modify these applied torques. Such adaptive behavior can compensate for non-ideal control and is well known from simple passive prostheses which could not function otherwise. It would be desirable to include these adaptations in the optimal control solutions, but this would require that the human body dynamics and its control are added to the system Eq (8). Such adaptation problems have been solved for sports equipment [30] and could be considered for prosthetics as well. The cost function would have to include a third objective, the user’s effort minimization, making the Pareto analysis more complicated.

During zero-energy operation, our proposed system generates net positive ankle work only during fast walking, about 1 J. It would be possible to increase this, by weighting the ankle tracking more heavily in the optimization objective, but this would increase tracking error at the knee in order to harvest more energy. The transfer of mechanical energy from knee to ankle is diminished by the motor heat and converter losses which consume almost all the energy that is harvested from the knee. Nevertheless, the proposed system, with its energy losses, still performs better than a single-joint system. We performed optimizations in which the knee was disconnected, to predict the performance of an active prosthetic ankle with the same controlled energy storage system. When the energy use was constrained to zero, the ankle tracking errors were 1.0, 2.2, and 3.9 degrees respectively, at slow, normal, and fast walking speed. Compared to the two-joint system (Table 4), performance of the single-joint system is significantly worse at normal and fast walking speed.

Previously, most research in this area was focused on developing single joint prostheses and used mechanical components, such as springs, for energy storage and energy transfer [e.g. [13, 18]]. An electromechanical system, as proposed in the present paper, has higher energy losses (as shown in Table 4), but it gives us the ability to control the energy flow, resulting in a semi-active system [21], where we have full control over joint torques at all times. Performance could be greatly improved if motors could be found with lower armature resistance, and/or a higher motor constant (Table 1), or if the efficiency of the power converters could be improved. Batteries have been used for energy regeneration [9], but these are less efficient than ultracapacitors and may not be able to absorb large bursts of power.

The fluctuations in stored energy were less than 40 J (Fig 3), suggesting that a smaller capacitor could have been used. However, a larger capacitor would allow activities where energy regeneration is not feasible, such as uphill walking or stair climbing. The capacitor would then have to be recharged during subsequent level walking activities by operating slightly to the left of the zero energy point in the Pareto diagram ((Fig 2)).

Optimal design parameters are an important result of this study. Gear ratios of about 275 were found to be optimal for both motors and for walking at all three speeds. Lower gear ratios would lead to larger motor torque, hence larger current which would generate more heat. Higher gear ratios are suboptimal also, because the effect of rotor inertia increases with the square of the gear ratio, making the system sluggish and requiring larger torques during the reversals of joint motion.

The optimal spring stiffness was larger in the ankle than in the knee. This was expected because passive prosthetic feet already use this principle. In the knee, passive stiffness resists flexion in the swing phase and this has prevented its use in passive prostheses. However, in combination with electric energy storage, passive knee stiffness was beneficial and improved the tracking for the zero-energy case. The spring resting positions *ϕ*_0_ were close to neutral in the ankle and about 30 degrees flexed in the knee. The leg will adopt this posture when the motors are off. Optimal spring stiffnesses depended on walking speed. In practice, it will be difficult for a user to change spring stiffness between activities. Through further simulations, we determined that performance of the system was not very sensitive to the spring parameters. This means that the same hardware can possibly be used for different activities. In future work, design optimization should be performed for other movement tasks, such as walking on slopes and stairs, and running. Other motor specifications should be considered as well. We speculate that it will be beneficial to use motors with a larger motor constant and lower resistance, which will lower the gear ratio and energy losses.

The current work is based on trajectory optimization with open loop control. While the open-loop control approach is valid for the purpose of design and feasibility analysis, an open-loop controlled system will likely not be robust with respect to disturbances. In a practical application, it will be necessary to translate the open loop controls into a sensor-based controller. For instance, optimal linear feedback control can be added by applying the linear quadratic regulator (LQR) method to a linear time-varying (LTV) system obtained through linearization of the system dynamics around the optimal trajectories [31].

## Conclusions

It is concluded that: (1) The proposed semi-active knee-ankle prosthesis can produce near-normal knee and ankle motion during walking without an external energy supply, and: (2) Optimal design parameters and control signals can be found for any human movement for which joint torque and motion trajectories are known.

## Supporting information

S1 File Matlab code (S1_File.m) to perform the optimizations. (M)

S1 Data Human gait data (S1_Data.mat), used by the optimization code. (MAT)
